# Impact of Endoscopic Lung Volume Reduction on Right Ventricular Myocardial Function

**DOI:** 10.1371/journal.pone.0121377

**Published:** 2015-04-09

**Authors:** Carmen Pizarro, Robert Schueler, Christoph Hammerstingl, Izabela Tuleta, Georg Nickenig, Dirk Skowasch

**Affiliations:** University Hospital Bonn, Department of Internal Medicine II, Cardiology, Pneumology and Angiology, Bonn, Germany; Clinica Universidad de Navarra, SPAIN

## Abstract

**Introduction:**

Endoscopic lung volume reduction (ELVR) provides a minimally invasive therapy for patients with severe lung emphysema. As its impact on right ventricular (R_t_V) function is undefined, we examined the extent of R_t_V functional changes following ELVR, as assessed by use of speckle tracking-based R_t_V deformation analysis.

**Methods:**

We enrolled 32 patients with severe emphysematous COPD scheduled for bronchoscopic LVR using endobronchial valves (Zephyr, PulmonX, Inc.), comprising 16 matched clinical responders and 16 non-responders. Echocardiography was conducted one day prior to ELVR and at an eight-week postprocedural interval.

**Results:**

Patients were predominantly of late middle-age (65.8±8.7yrs), male (62.5%) and presented advanced COPD emphysema (means FEV1 and RV: 32.6% and 239.1% of predicted, respectively). After ELVR, R_t_V apical longitudinal strain improved significantly in the total study cohort (-7.96±7.02% vs. -13.35±11.48%, p=0.04), whereas there were no significant changes in other parameters of R_t_V function such as R_t_V global longitudinal strain, TAPSE or pulmonary arterial systolic pressure. In responding patients, 6MWT-improvement correlated with a decrease in NT-proBNP (Pearson´s r: -0.53, p=0.03). However, clinical non-responders did not exhibit any R_t_V functional improvement.

**Discussion:**

ELVR beneficially impacts R_t_V functional parameters. Speckle tracking-based R_t_V apical longitudinal strain analysis allows early determination of R_t_V contractile gain and identification of clinical responsiveness.

## Introduction

Chronic obstructive pulmonary disease (COPD) is a common respiratory condition that will shortly constitute the third leading cause of mortality worldwide [1]. As a result of progressive limitations to airflow, a decrease in the number of small airways and emphysematous destruction of lung parenchyma, alveolar hypoxia and respiratory insufficiency arise. Right ventricular (RtV) dysfunction in COPD is the result of multifactorial mechanisms. Alveolar hypoxemia is the major cause of pulmonary vascular disease that follows two different ways of action: whereas acute hypoxemia induces hypoxic pulmonary vasoconstriction, chronic hypoxemia causes structural changes in the pulmonary vasculature, referred to as “remodelling” of the pulmonary vascular bed [2]. However, apart from hypoxemia-induced increase in pulmonary vascular resistance that finally leads to pulmonary hypertension (PH)-mediated right heart impairment, RtV dysfunction in COPD can also be observed in the absence of PH. Hilde et al. reported impaired RtV systolic function, RtV hypertrophy and dilation in COPD patients without PH as compared to healthy controls [3]. These additional mechanisms comprise, among others, systemic inflammation, endothelial dysfunction as well as lung hyperinflation.

As the degree of R_t_V dysfunction and presence of RtV failure determine prognosis in COPD [4], non-invasive imaging modalities for evaluation of R_t_V function are imperative. Right ventricular speckle tracking and two-dimensional visualization of RtV strain permit an accurate analysis of RtV deformation capabilities and is reported to predict future right heart failure [5,6].

In consideration of the progressive and wasting nature of COPD [7], new therapeutic modalities—complementing the established medical management of inhaler and anti-inflammatory therapy—have been proposed [8]. They primarily address the advanced emphysematous lung destruction in COPD that in turn is not responsive to medical treatment. Emphysema-induced dyspnoea arises from loss of elastic retraction, causing static and dynamic hyperinflation and consequently inefficiency of respiratory muscle activity [9]. Lung volume reduction surgery was studied in the National Emphysema Treatment Trial, which demonstrated, in a subset of patients, significant improvements in pulmonary function and exercise performance [10]. The concept guiding this approach is to remove damaged, overinflated tissue to alleviate the mechanical burden on the respiratory muscles and thereby reduce breathlessness. In view of the procedure’s associated increase in short-term mortality and morbidity [11], less invasive, bronchoscopically conducted methods to achieve lung volume reduction—without the complications accompanying surgery—have been developed. Endoscopic lung volume reduction (ELVR) comprises diverse techniques, of which endobronchial valve (EBV) implantation is the most studied [12,13].

The aim of the present study was to investigate the impact that ELVR by EBV placement has on right ventricular function, as measured by echocardiography and two-dimensional strain analysis, and to correlate the results with patient clinical outcomes.

## Methods

### Study population

A total of fifty-six patients with heterogeneous emphysema, admitted to the University Hospital Bonn, Germany, for ELVR by EBV placement, from January 2013 to January 2014, were evaluated for study participation. Consistent with VENT study criteria [12], patients were found eligible for the study if they met the following inclusion criteria: a heterogeneous emphysema distribution, a forced expiratory volume in one second (FEV1) of 15 to 45% of predicted, a residual volume (RV) ≥150% of predicted, a total lung capacity (TLC) ≥100% of predicted and a six-minute walk test distance (6MWTD) ≥140 m. The exclusion criteria for study enrolment comprised pre-existence of coronary artery disease, dilated cardiomyopathy and advanced congestive heart failure. On the day prior to ELVR and eight weeks thenceforth, a 6MWT was performed by serial measurement of two walks and use of a “best-of-two” approach [14]. Clinical responder status was defined by a postprocedural improvement in 6MWTD ≥25 meters [15]. Thirty-eight out of fifty-six patients (67.9%) showed clinical responsiveness to ELVR, whereas eighteen patients (32.1%) were clinical non-responders and represented the failure portion to ELVR. On the basis of demographic and preprocedural lung functional parameters, sixteen clinical non-responders to ELVR were consecutively compared to sixteen responders, resulting in a final study population size of thirty-two patients.

### Ethics statement

The study was approved by the medical ethics committee of Bonn and was conducted in accordance with the Declaration of Helsinki. All patients gave their written informed consent of participation.

### Follow up

Clinical and laboratory examinations were conducted at baseline and at 8-week interval post-ELVR. They comprised echocardiography and two-dimensional speckle tracking, pulmonary function testing according to the ERS guidelines [16] and laboratory measurement of plasma N-terminal pro-BNP (NT-proBNP) levels. Additionally, post-procedural chest X-ray at week 8 was conducted and compared to baseline thoracic computed tomography for the occurrence of pulmonary atelectasis and diaphragmatic movement.

### Procedure

We performed flexible bronchoscopy under moderate sedation with midazolam and propofol, permitting spontaneous breathing and without requirement for anaesthesiological assistance. Zephyr endobronchial valves (Pulmonx, Inc., Redwood City, Calif., USA) were placed unilaterally to isolate the target lobe. Target lobe was previously identified by computed tomography visualized severity of lobar emphysema and scintigraphically determined perfusional and ventilatory reduction. Prior to valve implantation, the Chartis Pulmonary Assessment System (Pulmonx, Inc., Redwood City, Calif., USA) was employed to assess the level of collateral ventilation (CV) [17]. Under the premise of CV-absence, ELVR by EBV was completed. All bronchoscopies were performed by the same experienced bronchoscopist (D.S.).

### Transthoracic echocardiography

Transthoracic two-dimensional echocardiography and colour tissue Doppler imaging (TDI) were obtained by experienced cardiac sonographers (R.S., C.H.) using a 2.5-MHz phased-array transducer and conventional equipment (Vivid 7, General Electric Medical Health, Waukesha, Wisconsin, USA; iE 33, Philips Medical Systems, Koninklijke N.V., Hamburg, Germany). With the patient in the left lateral decubitus position, echocardiography followed a standardized protocol, beginning with the parasternal position (long and short axis view) and then in the apical four, five, two and three chamber views. The pulmonary arterial systolic pressure (PASP) was estimated from the peak tricuspid regurgitation jet velocities. Echocardiographic recordings accorded with the recommendations of the American Society of Echocardiography [18].

### Two-dimensional speckle tracking echocardiography

**Fig 1:**
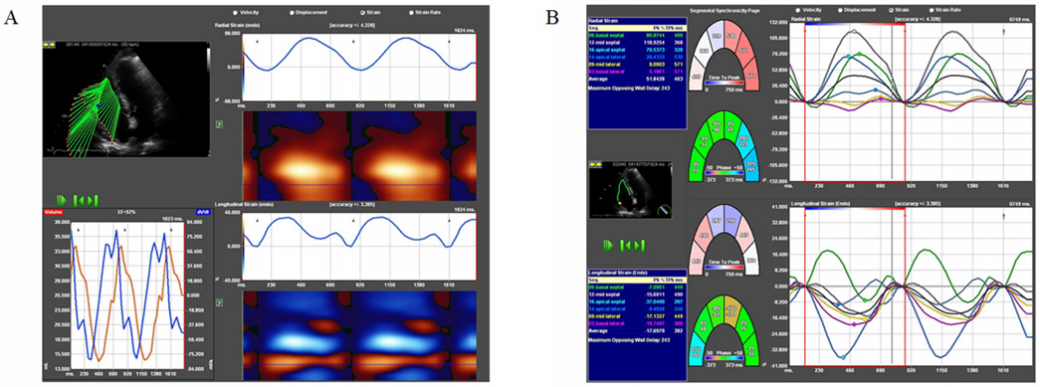
Speckle tracking derived visualization of right ventricular strain. (A) Upper left: In an apical four-chamber view, speckle artefacts in the right ventricle which are caused by refraction, reflection and scattering of echo beams, are tracked throughout the heart cycle. The visualization of their displacement allows deduction of the contractility of the right ventricular myocardium. Two different types of myocardial wall strains, the radial (upper right) and the longitudinal strain (bottom right), are illustrated over the cardiac cycle. (B) For the radial (upper half) and the longitudinal strain (lower half), division into myocardial regions—i.e. the apical, midventricular and basal segments—is performed. Additionally, an average value indicates global strain. Quantitative data and their correspondent graphical representation are colour-coded.

Speckle tracking was used to assess angle-independent myocardial deformation. An apical four-chamber view of the right ventricle was transferred to medical imaging software (TomTec Imaging Systems GmbH, Unterschleissheim, Germany). Beginning and ending at the tricuspidal annulus, the R_t_V endocardium was traced manually in the context of offline analysis, and was tracked with software support throughout two cardiac cycles. Apical, midventricular and basal segmentation was performed visually alongside the lateral free R_t_V wall, permitting global and regional longitudinal strain evaluation (Fig 1). Speckle tracking analysis was performed by versed cardiologists, blinded to hemodynamic and clinical information.

### Statistics

Descriptive statistics are presented as absolute numbers and percentages, means (±SD) or medians (range), when appropriate. In the case of categorical variables, Pearson’s χ^2^ test was used to test for associations; the unpaired t-test was applied to compare the means of continuous parameters. Correlations between variables were examined with Pearson correlation coefficient. Non-parametric testing by use of Kruskal-Wallis one-way analysis of variance was performed for evaluation of ordinal scaled variables. A p-value below 0.05, resulting from a two-tailed test, was considered statistically significant. Receiver operating characteristic (ROC) curve analysis for predicted probability was conducted for the RtV apical longitudinal strain; the correspondent area under the curve with its 95% CI was calculated. In 10 random patients, inter- and intraobserver reproducibility of strain measurement was tested by intraclass correlation coefficient with a coefficient ≥0.8 indicating a good reliability. All statistical analyses were performed by use of SPSS Statistics 22 software (IBM, Armonk, NY, USA).

## Results

The preinterventional demographic and clinical data of the 32 enrolled patients are presented in Table 1. The study population was predominantly male (62.5%, n = 20) and late middle-aged (65.8±8.7 years). The baseline pulmonary function testing evinced a mean FEV1 of 0.92 l (±0.25 l) in absolute terms and 32.6% (±8.0%) of the predicted value. The baseline residual volume accounted for 5.53 l (±1.05 l) and 239.1% (±52.4%) of predicted. The left lower lobe was targeted in a majority of patients (43.8%), followed by the right upper lobe (in 31.3%), the right lower lobe (in 18.7%) and the left upper lobe in the remaining 6.2%. The preprocedural echocardiographic parameters are summarised in Table 1.

**Table 1:** Patient demographic, clinical and echocardiographic characteristics at baseline, subdivided according to clinical responder status.

			All patients	Responders	Non-Responders	p-value
Subjects			32	16	16	
Age, years			65.8 ± 8.7	67.1 ± 8.7	64.6 ± 8.8	0.44
Male gender (n,%)			20 (62.5%)	10 (62.5%)	10 (62.5%)	1.00
Smoking status (n,%)	Current smoker		0 (0%)	0 (0%)	0 (0%)	0.64
	Former smoker		32 (100%)	16 (100%)	16 (100%)	
	Never smoker		0 (0%)	0 (0%)	0 (0%)	
Pack-years			40 (15–200)	40 (20–80)	35 (15–200)	0.69
FEV1, l			0.92 ± 0.25	0.90 ± 0.23	0.94 ± 0.28	0.72
FEV1, % predicted			32.60 ± 7.98	31.71 ± 6.28	33.48 ± 9.51	0.54
RV, l			5.53 ± 1.05	5.55 ± 1.00	5.50 ± 1.14	0.90
RV, % predicted			239.11 ± 52.41	235.29 ± 50.40	242.93 ± 55.73	0.69
RV/TLC ratio			74.11 ± 7.04	74.19 ± 5.82	74.03 ± 8.28	0.95
RV/TLC ratio, % predicted			185.00 ± 24.36	183.47 ± 24.80	186.53 ± 24.62	0.73
6MWTD, m			300 (140–600)	300 (140–390)	325 (140–600)	0.13
NT-proBNP, pg/ml			162.1 ± 119.2	161.3 ± 123.9	163.0 ± 118.5	0.97
Serum creatinine concentration, mg/dl			0.95 ± 0.44	1.01 ± 0.56	0.90 ± 0.28	0.49
Target lobe (n,%)	Right upper lobe		10 (31.3%)	4 (25.0%)	6 (37.5%)	0.60
	Right lower lobe		6 (18.7%)	4 (25.0%)	2 (12.5%)	
	Left upper lobe		2 (6.2%)	1 (6.2%)	1 (6.2%)	
	Left lower lobe		14 (43.8%)	7 (43.8%)	7 (43.8%)	
Antiobstructive and antiinflammatory medication use (n,%)	Short-acting beta_2_ agonist		20 (62.5%)	11 (68.8%)	9 (56.3%)	0.47
	Short-acting anticholinergic		22 (68.8%)	9 (56.3%)	13 (81.3%)	0.13
	Long-acting beta_2_ agonist		32 (100%)	16 (100%)	16 (100%)	*
	Long-acting anticholinergic		32 (100%)	16 (100%)	16 (100%)	*
	Inhaled glucocorticoids		30 (93.8%)	16 (100%)	14 (87.5%)	0.14
	Triple inhaler therapy		30 (93.8%)	16 (100%)	14 (87.5%)	0.14
	Systemic glucocorticoids		6 (18.8%)	1 (6.3%)	5 (31.3%)	0.07
	PDE-4 inhibitor (Roflumilast)		16 (50.0%)	7 (43.8%)	9 (56.3%)	0.48
Home oxygen therapy (n,%)			18 (56.3%)	8 (50.0%)	10 (62.5%)	0.48
Cardiovascular medication use (n,%)		Aspirin	10 (31.3%)	5 (31.3%)	5 (31.3%)	1.00
		Clopidogrel	1 (3.1%)	1 (6.3%)	0 (0%)	0.31
		Statin	9 (28.1%)	3 (18.8%)	6 (37.5%)	0.24
		ACE inhibitor	13 (40.6%)	8 (50.0%)	5 (31.3%)	0.28
		Angiotensin II receptor blocker	6 (18.8%)	3 (18.8%)	3 (18.8%)	1.00
		Beta-blocker	10 (31.3%)	4 (25.0%)	6 (37.5%)	0.45
		Calcium channel blocker	9 (28.1%)	5 (31.3%)	4 (25.0%)	0.69
		Diuretics	13 (40.6%)	7 (43.8%)	6 (37.5%)	0.72
Comorbidities (n,%)		Arterial hypertension	22 (68.8%)	12 (75.0%)	10 (62.5%)	0.45
		Diabetes mellitus	5 (15.6%)	1 (6.3%)	4 (25.0%)	0.14
		Unipolar depression	11 (34.4%)	4 (25.0%)	7 (43.8%)	0.26
Echocardiographic parameters						
LVEF, %			61.1 ± 10.9	61.1 ± 11.7	61.2 ± 10.5	0.97
Diastolic LV dysfunction		No diastolic LV dysfunction	4 (12.5%)	1 (6.3%)	3 (18.8%)	0.48
		Grade I	24 (75.0%)	13 (81.3%)	11 (68.8%)	
		Grade II	4 (12.5%)	2 (12.5%)	2 (12.5%)	
		Grade III	0 (0%)	0 (0%)	0 (0%)	
PASP, mmHg			29.0 ± 14.3	29.4 ± 15.6	28.6 ± 13.3	0.87
PASP (n,%)		<30mmHg	18 (56.3%)	12 (75.0%)	6 (37.5%)	0.05
		30 -<50mmHg	11 (34.4%)	3 (18.8%)	8 (50.0%)	
		50-<70mmHg	2 (6.3%)	0 (0%)	2 (12.5%)	
		≥70mmHg	1 (3.1%)	1 (6.3%)	0 (0%)	
TAPSE, mm			22.0 ± 5.3	20.8 ± 6.1	23.3 ± 4.2	0.17
2D global RV-Sl, %			-11.23 ± 6.47	-8.24 ± 6.25	-14.21 ± 5.32	0.01
2D apical RV-Sl, %			-7.96 ± 7.02	-5.39 ± 4.80	-10.54 ± 8.04	0.05
2D medial RV-Sl, %			-11.46 ± 7.16	-8.94 ± 5.43	-13.97 ± 7.94	0.06
2D basal RV-Sl, %			-14.72 ± 10.17	-10.48 ± 8.17	-18.96 ± 10.44	0.06

Data are presented as total number and percentage (in parentheses), mean ± SD or median and range (in parentheses).

* P-values not calculable

Abbreviations:

FEV1 = forced expiratory volume in 1 second

RV = residual volume

RV/TLC = residual volume/total lung capacity

CAT = COPD Assessment Test

6MWTD = 6 minute walk test distance

NT-proBNP = N-terminal pro-BNP

LVEF = left ventricular ejection fraction

LV = left ventricular

PASP = pulmonary arterial systolic pressure

TAPSE = tricuspid annular plane systolic excursion

2D global RV-Sl, % = two dimensional right ventricular global longitudinal strain

2D apical RV-Sl, % = two dimensional right ventricular apical longitudinal strain

At eight weeks, pulmonary function exhibited an increase in FEV1 of predicted by 4.11% (95%CI: +0.99%–+7.24%; p = 0.01) and 12.6%, in absolute and relative terms respectively. The mean residual volume decreased by an absolute 12.51% (95%CI: -32.08%–+7.05%; p = 0.20) of the predicted value, corresponding to a relative decrease of 5.23 percentage points. The RV/TLC ratio, used as a descriptive marker for air trapping and airways obstruction, revealed an absolute and relative decrease of 8.56% and 4.63% of predicted, respectively (95%CI: -17.27 –+0.14%; p = 0.05). Lung functional parameters as a function of 6MWT-guided clinical responsiveness are summarised in Table 2.

**Table 2:** Pulmonary function and echocardiographic outcomes at eight-week follow up.

	All patients			Responders			Non-Responders		
	Pre-ELVR	Post-ELVR	p-value	Pre-ELVR	Post-ELVR	p-value	Pre-ELVR	Post-ELVR	p-value
FEV1, l	0.92 ± 0.25	1.03 ± 0.35	0.01	0.90 ± 0.23	1.07 ± 0.41	0.04	0.94 ± 0.28	1.00 ± 0.30	0.22
FEV1, % predicted	32.60 ± 7.98	36.71 ± 11.57	0.01	31.71 ± 6.28	37.02 ± 10.87	0.03	33.48 ± 9.51	36.40 ± 12.57	0.18
RV, l	5.53 ± 1.05	5.30 ± 1.47	0.30	5.55 ± 1.00	5.27 ± 1.26	0.35	5.50 ± 1.14	5.33 ± 1.70	0.61
RV, % predicted	239.11 ± 52.41	226.60 ± 58.38	0.20	235.29 ± 50.40	221.19 ± 52.71	0.32	242.93 ± 55.73	232.00 ± 64.83	0.48
RV/TLC ratio	74.11 ± 7.04	71.06 ± 9.76	0.08	74.19 ± 5.82	71.48 ± 8.62	0.20	74.03 ± 8.28	70.65 ± 11.05	0.24
RV/TLC ratio, % predicted	185.00 ± 24.36	176.44 ± 25.40	0.05	183.47 ± 24.80	175.58 ± 22.50	0.15	186.53 ± 24.62	177.29 ± 28.74	0.20
CAT, points	22.7± 5.9	22.8 ± 7.3	0.89	22.5 ± 5.0	23.3 ± 8.1	0.67	22.9 ± 6.9	22.4 ± 6.6	0.73
6MWTD, m	302.50 ± 130.20	349.06 ± 127.86	0.02	267.50 ± 87.98	401.88 ± 74.56	<0.001	337.50 ± 156.79	296.25 ± 149.26	0.02
NT-proBNP, pg/ml	162.1 ± 119.3	178.6 ± 235.2	0.70	161.3 ± 123.9	111.6 ± 93.7	0.15	163.0 ± 118.5	245.6 ± 309.9	0.29
Serum creatinine concentration, mg/dl	0.95 ± 0.44	0.96 ± 0.44	0.78	1.01 ± 0.56	1.07 ± 0.57	0.24	0.90 ± 0.28	0.86 ± 0.22	0.27
Echocardiographic parameters									
LVEF, %	61.1 ± 11.1	60.1 ± 9.2	0.45	61.5 ± 12.1	60.7 ± 9.4	0.88	61.2 ± 10.5	59.6 ± 9.2	0.31
PASP, mmHg	29.0 ± 14.3	30.2 ± 14.6	0.50	29.4 ± 15.6	28.9 ±16.8	0.44	28.6 ± 13.3	31.5 ± 12.5	0.40
TAPSE, mm	22.0 ± 5.3	21.9 ± 5.3	0.92	20.8 ± 6.1	20.9 ± 4.9	0.85	23.3 ± 4.2	22.9 ± 5.6	0.82
2D global RV-Sl, %	-11.23 ± 6.47	-13.31 ± 6.53	0.19	-8.24 ± 6.25	-13.47 ± 7.61	0.06	-14.21 ± 5.32	-13.14 ± 5.49	0.45
2D apical RV-Sl, %	-7.96 ± 7.02	-13.35 ± 11.48	0.04	-5.39 ± 4.80	-13.55 ± 12.48	0.04	-10.54 ± 8.04	-13.15 ± 10.79	0.49
2D medial RV-Sl, %	-11.46 ± 7.16	-11.96 ± 6.92	0.78	-8.94 ± 5.43	-12.66 ± 6.63	0.11	-13.97 ± 7.94	-11.25 ± 7.35	0.34
2D basal RV-Sl, %	-14.72 ± 10.17	-11.13 ± 8.97	0.06	-10.48 ± 8.17	-8.15 ± 7.58	0.35	-18.96 ± 10.44	-14.10 ± 9.48	0.12

Data are presented as mean ± SD.

Abbreviations:

FEV1 = forced expiratory volume in 1 second

RV = residual volume

RV/TLC = residual volume/total lung capacity

CAT = COPD Assessment Test

6MWTD = 6 minute walk test distance

NT-proBNP = N-terminal pro-BNP

LVEF = left ventricular ejection fraction

PASP = pulmonary arterial systolic pressure

TAPSE = tricuspid annular plane systolic excursion

2D global RV-Sl, % = two dimensional right ventricular global longitudinal strain

2D apical RV-Sl, % = two dimensional right ventricular apical longitudinal strain

The two-dimensional speckle tracking evaluation of right ventricular deformation capabilities revealed significant improvement in R_t_V apical longitudinal strain (-7.96±7.02% vs. -13.35±11.48%, p = 0.04), whereas global and regional medial and basal R_t_V longitudinal strain did not change significantly over the follow-up period (Table 2).

**Fig 2:**
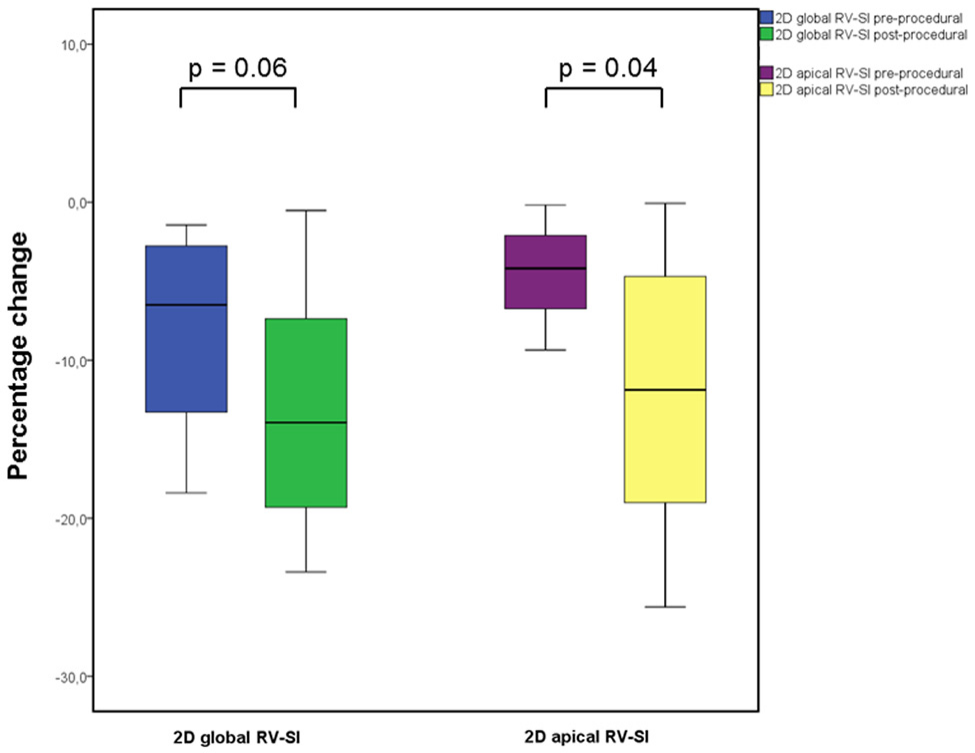
Pre- to post-procedural presentation of 2D global and apical RV-Sl in responding patients. Abbreviations: 2D global RV-Sl = two dimensional right ventricular global longitudinal strain 2D apical RV-Sl = two dimensional right ventricular apical longitudinal strain.

The 6MWTD increased an average 46.6 m (95%CI: +7.9 m—+85.3 m; p = 0.02) in the total study cohort. The clinical responders exhibited a mean 6MWTD improvement of 134.4 m (95%CI: +102.3 m—+166.5 m; p<0.001), a significant increase in R_t_V apical longitudinal strain (-5.39±4.80% vs. -13.55±12.48%, p = 0.04; Fig 2) and a statistical trend towards a postprocedural improvement in R_t_V global longitudinal strain (-8.24±6.25% vs. -13.47±7.61%, p = 0.06). In contrast, the global and regional R_t_V deformation properties remained unchanged in the case of the clinical non-responsiveness (Table 2).

Intra- and interobserver reliability analysis for speckle tracking- based strain data showed a good reproducibility. For the apical, medial, basal and global right ventricular strain, the interobserver difference was 1.93±4.05, 1.84±3.73, 0.54±5.71 and 0.64±3.12 of the mean strain for both observers, respectively. The correspondent intraclass correlation coefficient of interobserver variability was 0.86 (95%CI: 0.53–0.97, p<0.01), 0.80 (95%CI: 0.27–0.95, p<0.01), 0.89 (95%CI: 0.55–0.97, p<0.01) and 0.92 (95%CI: 0.68–0.98, p = 0.001), respectively. For intraobserver repeatability, the intraclass correlation coefficients for the apical, medial, basal and global right ventricular strain accounted for 0.99 (95%CI: 0.96–0.99, p<0.001), 0.93 (95%CI: 0.73–0.98, p<0.001), 0.96 (95%CI: 0.82–0.99, p<0.001) and 0.92 (95%CI: 0.66–0.98, p = 0.001), respectively.

Pulmonary arterial systolic pressure (PASP), as a conventional right ventricular functional parameter, remained statistically unchanged pre- to post-procedurally. Biventricular end-diastolic and end-systolic measured dimensions, stroke volumes and their correspondent indices did not differ pre-procedurally between the clinically responding and non-responding group (Table 3). Post-procedurally, a significant difference in the left and right ventricular stroke volume indices (p = 0.04 and <0.01, respectively), as well as in the right ventricular end-systolic volume index (p = 0.03) was observed by comparison of both groups. In case of clinical responsiveness, pre- to post-postprocedural comparison revealed a significant gain in right ventricular stroke volume and stroke volume index (p = 0.007 and p = 0.006, respectively), while neither the non-responding subgroup, nor the total study collective per se offered significant ELVR-mediated changes.

**Table 3:** Volumetric data as a function of clinical responder status.

	All patients	Responders	Non-Responders	p-value
Pre-ELVR				
Left ventricular end-diastolic volume, ml	82.6 ± 33.7	88.3 ± 40.9	76.9 ± 24.4	0.34
Left ventricular end-diastolic volume index, ml/m^2^	44.8 ± 17.2	48.2 ± 21.9	41.4 ± 10.4	0.27
Left ventricular end-systolic volume, ml	33.3 ± 21.7	35.9 ± 26.4	30.6 ± 16.1	0.50
Left ventricular end-systolic volume index, ml/m^2^	17.9 ± 11.5	19.7 ± 14.5	16.3 ± 7.5	0.41
Left ventricular stroke volume, ml	49.3 ± 17.2	52.4 ± 21.2	46.3 ± 12.0	0.32
Left ventricular stroke volume index, ml/m^2^	26.8 ± 8.6	28.6 ± 10.8	25.1 ± 5.6	0.27
Right ventricular end-diastolic volume, ml	40.2 ± 19.5	37.9 ± 17.7	42.6 ± 21.5	0.50
Right ventricular end-diastolic volume index, ml/m^2^	21.3 ± 8.8	20.2 ± 7.6	22.5 ± 9.9	0.46
Right ventricular end-systolic volume, ml	20.7 ± 12.1	19.7 ± 12.9	21.6 ± 11.5	0.67
Right ventricular end-systolic volume index, ml/m^2^	10.9 ± 5.8	10.6 ± 6.4	11.4 ± 5.4	0.68
Right ventricular stroke volume, ml	19.6 ± 10.7	18.1 ± 8.7	21.0 ± 12.5	0.45
Right ventricular stroke volume index, ml/m^2^	10.3 ± 4.9	9.6 ± 3.6	11.1 ± 6.0	0.40
Post-ELVR				
Left ventricular end-diastolic volume, ml	81.7 ± 30.6	89.5 ± 33.7	73.8 ± 25.9	0.15
Left ventricular end-diastolic volume index, ml/m^2^	44.4 ± 14.7	48.9 ± 16.8	39.9 ± 11.1	0.08
Left ventricular end-systolic volume, ml	34.3 ± 19.4	36.6 ± 20.9	32.0 ± 18.0	0.51
Left ventricular end-systolic volume index, ml/m^2^	18.5 ± 9.5	20.0 ± 10.8	17.0 ± 8.2	0.39
Left ventricular stroke volume, ml	47.3 ± 16.7	52.9 ± 18.2	41.8 ± 13.4	0.06
Left ventricular stroke volume index, ml/m^2^	25.9 ± 8.3	29.0 ± 9.0	22.9 ± 6.4	0.04
Right ventricular end-diastolic volume, ml	41.5 ± 15.2	42.4 ± 14.4	40.6 ± 16.4	0.74
Right ventricular end-diastolic volume index, ml/m^2^	22.3 ± 6.6	22.9 ± 5.9	21.6 ± 7.4	0.59
Right ventricular end-systolic volume, ml	21.0 ± 9.4	17.9 ± 7.7	24.1 ± 10.1	0.06
Right ventricular end-systolic volume index, ml/m^2^	11.2 ± 4.3	9.5 ± 3.3	12.8 ± 4.6	0.03
Right ventricular stroke volume, ml	20.5 ± 9.3	24.5 ± 9.5	16.5 ± 7.3	0.01
Right ventricular stroke volume index, ml/m^2^	11.1 ± 4.6	13.4 ± 4.5	8.8 ± 3.3	<0.01

Data are presented as mean ± SD.

Abbreviations:

ELVR = endoscopic lung volume reduction

**Fig 3:**
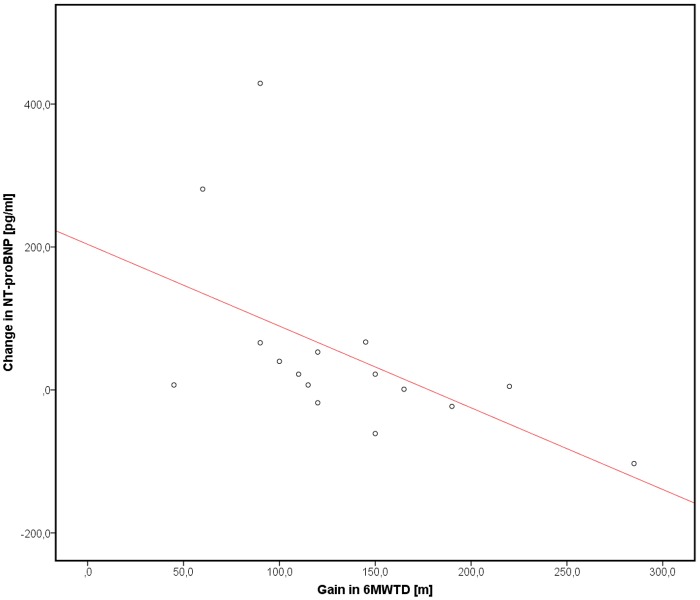
Changes in 6-minute walk test distance and NT-proBNP levels in clinical responders to endoscopic lung volume reduction at eight-week follow up. Abbreviations: 6MWTD = 6 minute walk test distance, NT-proBNP = N-terminal pro-BNP.

**Fig 4:**
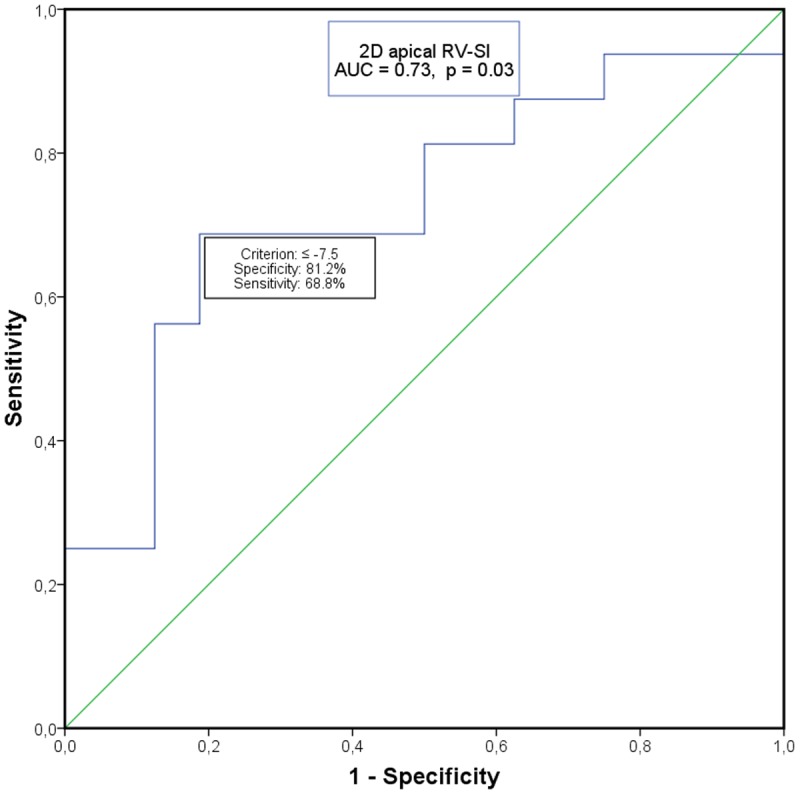
Receiver operating characteristic (ROC) cure analysis of 2D apical RV-Sl for the identification of patients responding to endoscopic lung volume reduction by endobronchial valve placement. Abbreviations: 2D apical RV-Sl = two dimensional right ventricular apical longitudinal strain**,** AUC = Area under the curve.

Complete atelectasis with lobar exclusion and diaphragmatic movement occurred in 56.3% of total cohort (responding subgroup: 62.5%, non-responding subgroup: 50.0%). Overall, laboratory testing revealed a non-significant relative mean increase in NT-proBNP of 10.2% (161.1±119.2 pg/ml vs. 178.6±235.2 pg/ml). In view of the established interaction between renal function and NT-proBNP-levels, we tested the correlation between serum creatinine concentration and NT-proBNP that presently permitted exclusion of an interparametral correlation (Pearson´s r: -0.11, p = 0.56). In the clinical responder subgroup, the increase in the 6MWTD was significantly associated with a reduction of the NT-proBNP-level (Pearson´s r: -0.53, p = 0.03, Fig 3), that in turn occurred independently of R_t_V strain adaptations. Receiver operating characteristic (ROC) curve analysis of RtV apical longitudinal strain for predicted probability of response to ELVR evinced an area under the curve of 0.73 (95%CI: 0.55–0.91 m; p = 0.03), indicating an overall fair accuracy. A cut-off value of -7.5 showed a specificity of 81.2% and a sensitivity of 68.8% (Fig 4). Neither pulmonary function nor echocardiographic changes manifested dependency on treated lobe.

Complications were limited to post-procedural occurrence and comprised early exacerbation of COPD in 9.4% of patients (n = 3), resolving under medical treatment without need for valve removal. Development of pneumothorax occurred in 4 patients (12.5%) and resolved adequately by chest tube implantation. No other kind of complications were observed.

## Discussion

This study analysed the impact of ELVR by EBV placement on global and regional R_t_V dysfunction and identified ELVR-mediated R_t_V improvement. R_t_V apical longitudinal strain might be a valuable tool to evaluate short-term RtV functional amelioration and clinical responsiveness to ELVR.

The course of advanced emphysematous COPD is sustainably complicated by cardiac complications on the right heart side, with alterations of right ventricular function being predictive of patient´s outcome [19,20]. Numerous efforts to identify the optimal parameter to evaluate systolic and diastolic R_t_V function have been undertaken. Speckle tracking echocardiography was introduced by Reisner and Leitman in 2004 [21,22] and represents by now an established imaging modality. Acoustic reflections, generated by ultrasound interrogation of tissue, allow a semiautomated measurement of myocardial deformation properties. The resulting dimensionless strain quantifies global and regional ventricular contractility. In contrast to tissue Doppler imaging, speckle tracking is independent of the angle of the ultrasound beam, facilitating an easy and robust application [6]. In the field of newly diagnosed pulmonary arterial hypertension (WHO group 1), Sachdev et al. identified R_t_V longitudinal strain and strain rate to be predictive of future right heart failure, clinical worsening and mortality [23]. In a subsequent study, he demonstrated consistence of results by serial strain assessment even after initiation of medical PAH-treatment, emphasizing the prognostic utility of strain measurement [24].

In COPD, the mechanisms leading to RtV dysfunction are diverse. They comprise a much broader spectrum than sole pulmonary hypertension caused by hypoxic pulmonary vasoconstriction and pulmonary vascular remodelling [2]. These additional mechanisms comprehend lung hyperinflation, endothelial dysfunction and systemic inflammation, all of them able to exert a direct negative impact on the right ventricle [3]. In a COPD study population that comprised both confirmed and excluded pulmonary hypertension COPD patients, Hilde et al. showed that reduced RtV systolic function, RtV hypertrophy and dilation were even present in COPD patients without pulmonary hypertension as compared to healthy controls [3]. In this line of thought, the presently described ELVR-mediated improvement in exercise capacity—measured by 6MWT-perfomance—and RtV function would be ascribable to an improvement in lung hyperinflation that in turn has been correlated to right and left ventricular volumetric conditions. Jörgensen et al. [25] compared 13 patients with severe emphysema to matched healthy controls and objectified impairment in RtV and LV performance status in case of severe emphysema. The compromised cardiac performance was ascribed to a preload reduction, primarily caused by intrathoracic hypovolemia in consequence of pulmonary hyperinflation. Transferring these considerations to our study, we additionally measured biventricular end-diastolic and end-systolic dimensions, as well as biventricular stroke volumes. Clinical responder status was associated with a significant gain in RtV stroke volume (p<0.01) and stroke volume index (p<0.01). Simultaneously, though not significantly, R_t_V end-diastolic volume index increased by an absolute 2.75±7.17ml/m^2^ and a relative 13.6%. In case of surgically performed lung volume reduction, the response of right ventricular performance was investigated by Mineo et al. [26]. Twelve patients undergoing bilateral lung volume reduction surgery (LVRS) were assessed preoperatively and six months afterwards: they showed a significant increase in right ventricular stroke volume both at rest and during exercise.

The effect of endoscopic lung volume reduction (ELVR) on RtV function remains undetermined. ELVR by EBV placement represents an emerging, minimally invasive therapeutic modality to achieve reduction of overinflated, functionless pulmonary regions without the LVRS-associated perioperative complications [27]. Theoretically, the diminishment of emphysematous tissue is expected to ameliorate RtV performance through different mechanisms. On the one hand, the elimination of damaged areas decreases overall hyperinflation, improves rib cage and diaphragm mechanics and thereby renders more effective the work of breathing. By reducing alveolar hypoxia, hypoxic pulmonary vasoconstriction is diminished and the ventilation/perfusion ratio improves. On the other hand, a reduction in the hyperinflation-driven compression both of the vascular bed and of the ventricular relaxation, as well as an amelioration of the polycythemia-caused hyperviscosity also contributes to RtV relief.

The impact of ELVR by EBV on pulmonary hypertension in 6 patients with severe emphysematous COPD and established pulmonary hypertension was recently reported by Eberhardt et al. [28]. Right heart catheterization was performed prior to and at 90 days after ELVR. In 5 out of 6 patients hemodynamics improved with a decrease in mean pulmonary artery pressure and pulmonary capillary wedge pressure and gain in cardiac index. By contrast, our study collective comprised only a minority of PH-patients (14 patients (43.8%)) and echocardiographically-measured pulmonary arterial systolic pressure (PASP) elevation was only modest and did not change substantially post-ELVR, pointing at other mechanisms responsible for RtV functional and clinical improvement, as indicated previously.

In the presently studied COPD collective, the mean baseline RtV global longitudinal strain was -11.2±6.5% and differed considerably from the reference values in healthy subjects that have recently been described to average -27±2% [29]. This discrepancy is primarily attributable to the impairment in RtV systolic function that emphysematous COPD is associated with. In a COPD study population that comprised both confirmed and excluded pulmonary hypertension COPD subgroups [3], longitudinal strain assessment—though performed from the basal RtV free wall—was -22% and -18% in case of PH-absence and PH-presence, respectively, versus -31% in healthy controls. Moreover, strain correlated significantly with forced expiratory volume in 1sec (FEV1). In concrete terms, FEV1 was 46% of predicted in the COPD-subgroup without pulmonary hypertension and consequently considerably higher than in our study population (FEV1% predicted: 32.6%).

We presently identified R_t_V apical longitudinal strain to be the best correlative marker of clinical responsiveness. Its value in predicting the presence of pulmonary hypertension has been investigated by Lopez-Candales et al., who analysed longitudinal strain and velocity data in PH patients, compared them to healthy controls and ascribed the highest degree of PH predictability to alterations in apical longitudinal R_t_V strain [30]. Moreover, its impairment has been demonstrated repeatedly to be predictive of R_t_V heart failure, clinical deterioration and mortality [23,30]. Against the present background of our unobserved ELVR-mediated changes in conventional R_t_V functional parameters, such as PASP or TAPSE, the diagnostic advantage of strain analysis appears to be its early detection of functional adaptations post-ELVR, adaptations that might possibly precede changes in the above-mentioned traditional echocardiographic measures. Increasing evidence suggests that a post-ELVR clinical performance status enables a more feasible evaluation of procedural benefit than mere pulmonary function testing [31]. Here, we present as statistically significant a gain in FEV1 and a decrease in the RV/TLC ratio that, however, occurred independently of exercise improvement and amelioration of myocardial function, and which conditioned our decision to assess clinical responsiveness by changes in the 6MWT.

On the whole and in comparison with the responding subgroup, clinical non-responders appeared to be “fitter” at baseline in terms of age (64.6 vs. 67.1 years), 6MWTD (325 vs. 300m) and echocardiographic performance (PASP, global and regional longitudinal RtV strain). After ELVR, the global RtV-Sl strain increased in the responding subgroup by an absolute 5.2% and a relative 63.5%. However,—in case of clinical non-responsiveness—the global RtV-Sl strain even worsened by 1.07% and 7.5% in absolute and relative terms, respectively, that was additionally accompanied by a lack in PFT parameter improvement. In the light of the above, though baseline global RtV-Sl strain differed significantly between groups—a fact that seems to be indicative of major baseline RtV functional reserves in non-responders—, the opposite global strain development after ELVR illustrates the basic differing treatment response to ELVR between groups.

There are several limitations to this study that should be addressed. Given the small number of patients examined, the generalization of results is somewhat restricted. Although matching was performed to attain demographic and lung functional group homogeneity, selection bias on the basis of baseline echocardiographic parameters cannot be excluded. Due to the lack of a third subgroup comprising COPD patients without ELVR, estimation of R_t_V function alteration in ELVR-naive COPD is limited. To test for correlations with PASP, invasive hemodynamic data would have been required, but these presently were not obtained. Nonetheless, the accuracy of echocardiographically-determined pulmonary arterial pressures has repeatedly been demonstrated, such as by Hammerstingl and colleagues, who ascertained non-invasive, echocardiographic assessment inclusive of R_t_V speckle tracking analysis to be of reliable value for estimating PH, with non-invasively measured PASP correlating significantly with invasively determined mean pulmonary arterial pressure [32]. In order to additionally capture R_t_V functional adaptations that are driven by ELVR-mediated changes in chronic-hypoxemia caused pulmonary vascular remodelling, complementary long-term follow-up of our study cohort would be of additional value.

In conclusion, the non-invasive evaluation of speckle-tracking-based R_t_V longitudinal function permits an early estimation of the beneficial effects of ELVR on R_t_V contractility and the differentiation of clinical responder status.
